# Race/Ethnicity and Self-Reported Levels of Discrimination and Psychological Distress, California, 2005

**DOI:** 10.5888/pcd9.120042

**Published:** 2012-10-18

**Authors:** DeAnnah R. Byrd

## Abstract

**Introduction:**

Little is known about the relationship between discrimination and distress among multiple racial groups because previous studies have focused primarily on either blacks or Asian Americans. The objective of this study was to assess the association between self-reported experiences of racial discrimination and symptoms of psychological distress among 5 racial/ethnic groups in California.

**Methods:**

I used data from the 2005 California Health Interview Survey describing an adult sample of 27,511 non-Hispanic whites, 8,020 Hispanics, 1,813 non-Hispanic blacks, 3,875 non-Hispanic Asians, and 1,660 people of other races/ethnicities. The Kessler 6-item Psychological Distress Scale determined symptoms of psychological distress. I used a single-item, self-reported measure to ascertain experiences of racial discrimination.

**Results:**

Reports of racial discrimination differed significantly among racial groups. Self-reported discrimination was independently associated with psychological distress after adjusting for race/ethnicity, age, sex, education level, employment status, general health status, nativity and citizenship status, English use and proficiency, ability to understand the doctor at last visit, and geographic location. The relationship between discrimination and psychological distress was modified by the interaction between discrimination and race/ethnicity; the effect of discrimination on distress was weaker for minority groups (ie, blacks and people of other races/ethnicities) than for whites.

**Conclusion:**

Self-reported discrimination may be a key predictor of high levels of psychological distress among racial/ethnic groups in California, and race appears to modify this association. Public health practitioners should consider the adverse effects of racial discrimination on minority health.

## Introduction

For many racial/ethnic minorities, the experience of prejudice and discrimination is part of everyday life. In 1 study, 80% of respondents reported having experienced racial discrimination at some point in their lives (1). Studies suggest that being treated differently because of one’s race/ethnicity can adversely affect overall health and well-being (2,3). Results from studies of racial discrimination and mental health indicate that higher levels of discrimination are associated with poorer mental health status (4,5). Mental health status includes a range of mental health outcomes, such as anxiety, depression and depressive symptoms, other psychiatric problems, and emotional and psychological distress.

Studies have examined the relationship between racial discrimination and psychological distress. For example, a review by Williams and Mohammed (5) references several cross-sectional studies that found a positive association between discrimination and psychological distress. However, most of these studies have used samples outside of the United States, including the United Kingdom and Sweden (6–8). Moreover, existing work and earlier studies of discrimination have disproportionately focused on blacks or Asian Americans; as a result, little is known about the discrimination–distress relationship among members of other ethnic minority groups (9–12).

On the basis of previous research, I hypothesized that minorities would report higher levels of discrimination than whites and that self-reported discrimination would be positively associated with symptoms of psychological distress (5). My final hypothesis was that the association between self-reported discrimination and psychological distress symptoms would differ by race/ethnicity, specifically, that the effect of self-reported discrimination on distress would be stronger among minorities than among whites. I posed this hypothesis for 2 reasons. First, ethnic identity may have a qualitatively different meaning for minorities than for whites. For example, minorities may view their race/ethnicity as a central part of their identity, whereas whites do not because they are placed at the higher end of a socially stratified racial hierarchy. Second, identity-relevant stressors that threaten a salient and central part of one’s identity may be harmful (13). Therefore, racial discrimination is likely a greater identity-relevant stressor for racial minorities and may have a deleterious effect on their psychological well-being. Whites tend to identify less with their ethnicity and thus may not experience discrimination or its effects as an identity-relevant stressor. The objective of this study was to assess the association between self-reported experiences of racial discrimination and symptoms of psychological distress among 5 racial/ethnic groups in California.

## Methods

### Study sample

The study sample was taken from the 2005 adult California Health Interview Survey (CHIS), one of the largest state health surveys in the United States. CHIS is a population-based, random-digit–dialed telephone survey administered biannually since 2001 and is approved by the institutional review board of the University of California, Los Angeles (UCLA). It collects information for all age groups on health status, health conditions, health-related behaviors, health insurance coverage, access to health care services, and other health-related issues for California’s population (14,15).

 At total of 43,020 adults living in households in California completed the CHIS 2005 survey for an overall response rate of 26.9%. The CHIS response rate is comparable to response rates of other scientific telephone surveys in California, such as the 2005 California Behavioral Risk Factor Surveillance System (BRFSS) survey, which had an overall response rate of 29.2% (14,15). My study used a sample of 42,879 respondents, comprising 27,511 non-Hispanic whites, 8,020 Hispanics, 1,813 non-Hispanic blacks, 3,875 non-Hispanic Asians, and 1,660 people of other races/ethnicities (eg, native Hawaiian/Pacific Islanders, people of mixed races). I excluded 141 respondents because of missing data.

To produce population estimates using the CHIS data, I used survey-supplied weights. The age range of California adult respondents was 18 to 45 years, and the average age was 44. The weighted sample was almost equally divided between men and women. Most respondents were white (48.6%), college graduates (32.5%), and employed (68.6%). More than 80% of respondents rated their general health status as excellent, very good, or good, and 83.8% reported having health insurance coverage.

### Measures

Symptoms of psychological distress were assessed using the Kessler 6-item Psychological Distress Scale (K6), which is a generalized measure of past-month distress (Cronbach α = .83) (16). Each question began with, “About how often during the past 30 days did you feel . . .” followed by a symptom: “nervous,” “hopeless,” “restless or fidgety,” “so depressed that nothing could cheer you up,” “everything was an effort,” and “worthless.” Each question had 5 possible responses ranging from 0 being “none of the time” to 5 being “all of the time.” Items were summed to obtain a total score ranging from 0 to 24. The K6 has been used in population surveys to measure generalized distress and to detect psychiatric disorders (17,18), although it does not assess a person’s need for treatment or define psychiatric disorders in a population-based sample. The scale does allow researchers to rank respondents on a continuum of reported distress levels.

Racial discrimination was measured with 1 question: “Thinking about your race or ethnicity, how often have you felt treated badly or unfairly because of your race or ethnicity?” The response options were 1 being “never,” 2 being “rarely,” 3 being “sometimes,” 4 being “often,” or 5 being “all of the time.” This question is similar to one used in a previous study (19). I calculated the average level of discrimination for each racial group by taking the sum of all 5 response categories and dividing by the total number of respondents, yielding the frequencies in the discrimination scores reported. Next, I obtained the mean discrimination scores for each racial/ethnic group, which ranged from 1 to 5. I then used linear regression to identify whether there were significant racial/ethnic differences in mean discrimination scores.

I derived a single measure of nativity and citizenship status by combining the response categories for 2 questions: “In what country were you born?” and “Are you a citizen of the United States?” I also assessed English use and proficiency with the question, “Would you say you speak English?” with the following options for responses: “I speak only English,” “I speak English very well or well,” “I do not speak English well,” or “I do not speak English at all.” To control for acculturative stress and immigration-related factors, I used the CHIS survey question, “The last time you saw a doctor, did you have a hard time understanding the doctor because you and the doctor spoke different languages?”

Covariates were age, sex, education level, employment status, general health status, health insurance status, and geographic location. These variables are associated with psychological distress and discrimination among racial/ethnic minorities (20,21).

### Statistical analysis

Analyses began with simple regression of discrimination on psychological distress symptoms. Having found a significant bivariate association between these 2 variables, I then controlled for several covariates. Ordinary least squares was used to test the effect of discrimination on distress, adjusting for covariates in the model. Finally, to determine whether the effect of discrimination on distress varied by race/ethnicity, I tested the overall differences in the relationship between discrimination and distress for whites (reference category), Hispanics, blacks, Asians, and people of other races using a Wald test. I also determined whether there were differences by race/ethnicity in the association between racial discrimination and psychological distress by testing the individual interaction coefficients (ie, racial differences in the discrimination–distress slope) using a *t* test.

All analyses were conducted by using Stata version 11.0 (StataCorp LP, College Station, Texas) to account for the complex sampling design, and all results were weighted to adjust for nonresponse and to allow estimates to be representative of the target population in California (14,15).

## Results

More than one-fourth (25.7%) of the weighted sample reported rarely experiencing racial discrimination, and the average K6 scores, indicating level of distress, ranged from 1.5 to 1.7 for each racial group (Table 1). Average reports of discrimination varied by race/ethnicity; all nonwhite racial/ethnic groups reported significantly higher levels than whites, who reported an average level of discrimination of 1.5 compared with 1.8 for Hispanics, 2.6 for blacks, 1.9 for Asians, and 1.9 for people of other races/ethnicities (includes native Hawaiian/Pacific Islanders and people of mixed races) (data not shown). The greatest difference in average reports of discrimination was between whites and blacks (β = −1.19, *P* < .001), meaning that blacks on average reported a 1.19% higher level of discrimination compared with whites. Blacks also reported a significantly higher average level of discrimination than all other nonwhite racial/ethnic groups (data not shown); the mean difference in reports of discrimination between Asians and respondents of other races/ethnicities was not significant (*t* = 0.47, *P* = .64). Linear regression model 1 (Table 2) shows a positive relationship between self-reported levels of racial discrimination and symptoms of psychological distress (β = 0.14, *P* < .001). This relationship remains significant after adjusting for other covariates (*t* = 20.55, *P* < .001), excluding health insurance status, which was not significant (model 2). For each 1-unit change in self-reported level of discrimination, there was an increase in the K6 score of 0.14, controlling for race/ethnicity, age, sex, education level, employment status, general health status, nativity and citizenship status, English use and proficiency, ability to understand doctor at last visit, and geographic location. Overall, this model explains 15% (*r* ^2^ = .149) of the variation in symptoms of psychological distress.

**Table 1:** Demographic Characteristics of California Adults, by Race/Ethnicity, California Health Interview Survey, 2005^a^

Characteristic	Non-Hispanic White (n = 12,797,218)	Hispanic (n = 8,131,939)	Non-Hispanic Black (n = 1,468,868)	Non-Hispanic Asian (n = 3,272,118)	Other Races/Ethnicities (n = 621,137)	Total (N = 26,291,280)
**Kessler 6-item Psychological Distress Scale score^b^, mean (95% CI)**	1.5 (1.52-1.54)	1.6 (1.57-1.62)	1.7 (1.61-1.71)	1.6 (1.53-1.58)	1.7 (1.65-1.76)	1.6 (1.56-1.57)
**Age, y, mean (95% CI)**	48.6 (48.46-48.83)	38.1 (37.89-38.38)	44.0 (43.16-44.82)	43.9 (43.34-44.38)	41.8 (40.89-42.63)	44.4 (44.34-44.41)
**Sex**						
Male	48.8	51.0	45.4	46.8	47.7	49.0
**Education level**						
Less than high school	5.2	38.6	8.4	9.9	10.4	16.4
High school graduate	25.8	28.5	32.1	19.8	32.0	26.4
Some college	27.2	20.5	34.7	18.9	34.7	24.7
College graduate or higher	41.7	12.4	24.7	51.4	23.0	32.5
**Employed**	66.7	71.9	67.5	68.0	69.9	68.6
**General health status**						
Excellent	25.3	14.6	20.7	17.9	18.4	20.6
Very good	36.1	21.2	28.2	30.5	31.4	30.2
Good	25.2	37.3	31.1	30.4	31.1	30.0
Fair	9.9	22.4	14.9	16.0	13.9	14.9
Poor	3.6	4.5	5.1	5.2	5.3	4.2
**Nativity and citizenship status**						
US-born citizen	90.9	42.4	94.2	20.1	92.3	67.3
Foreign-born citizen	5.9	17.9	3.6	51.8	4.7	15.2
Foreign-born noncitizen	3.2	39.7	2.3	28.1	3.0	17.5
**English use and proficiency**						
English speaker	88.0	19.1	90.4	20.6	79.6	58.3
Speaks English very well	11.7	42.7	9.2	55.3	20.3	26.8
Poor English or non-English speaker	0.3	38.2	0.3	24.1	0.2	15.0
**Understanding doctor at last visit**						
Understood doctor; he/she spoke the same language	98.8	95.7	98.3	97.6	98.1	97.6
Did not understand doctor; he/she spoke a different language	1.2	4.3	1.7	2.4	1.9	2.4
**Lives in a metropolitan area^c^**	95.9	99.3	99.8	99.7	95.2	97.6
**Insured**	91.9	70.1	87.1	85.0	83.6	83.8
**Self-reported experiences of discrimination**						
Never	66.3	49.4	15.2	40.2	42.8	54.4
Rarely	24.2	24.5	27.3	32.9	28.8	25.7
Sometimes	7.9	21.0	40.2	23.3	22.0	16.0
Often	1.1	4.2	12.8	2.5	5.3	3.0
All of the time	0.5	1.0	4.5	1.0	1.1	0.9

Abbreviation: CI, confidence interval.

^a^ Estimates are weighted to account for the sampling design. All data are percentages unless otherwise indicated. Because of rounding, percentages may not total 100.

^b^ Score range, 0-24.

^c^ Reference category is participants who live in a nonmetropolitan area.

**Table 2:** Nested Ordinary Least Squares Models of the Effects of Discrimination on Psychological Distress Among California Adults, California Health Interview Survey, 2005 (N = 26,291,280)^a^

Variable	Model 1, Discrimination Only			Model 2, Discrimination and Covariates		
	Β (SE)		*P* Value^b^	Β (SE)		*P* Value^b^
**Experiences of racial discrimination**	0.14 (0.07)		<.001	0.14 (0.01)		<.001
**Race/ethnicity**						
Non-Hispanic white	NA	NA		1 [Reference]	1 [Reference]	
Hispanic				−0.11 (0.02)		<.001
Non-Hispanic black				−0.11 (0.03)		<.001
Non-Hispanic Asian				−0.07 (0.02)		.001
Other races/ethnicities				0.03 (0.03)		.27
**Age, y**	NA	NA		−0.01 (0)		<.001
**Sex**						
Female	NA	NA		1 [Reference]	1 [Reference]	
Male				−0.07 (.01)		<.001
**Education**						
College graduate or higher	NA	NA		1 [Reference]	1 [Reference]	
Less than high school				0.16 (0.02)		<.001
High school graduate				0.07 (0.01)		<.001
Some college				0.05 (0.01)		<.001
**Employment status**						
Unemployed	NA	NA		1 [Reference]	1 [Reference]	
Employed				−0.12 (0.01)		<.001
**General health status fair or poor^c^**	NA	NA		−0.45 (0.01)		<.001
**Nativity and citizenship status**						
US-born citizen	NA	NA		1 [Reference]	1 [Reference]	
Foreign-born citizen				−0.02 (0.02)		.21
Foreign-born noncitizen				−0.07 (0.02)		<.001
**English use and proficiency**						
Speaks only English	NA	NA		1 [Reference]	1 [Reference]	
Speaks English very well				0 (0.01)		.74
Poor English or non-English speaker	NA	NA		−0.04 (0.02)		.06
**Did not understand doctor; spoke a different language**	NA	NA		0.19 (0.04)		<.001
**Lives in a metropolitan area^d^**	NA	NA		−0.01 (0.02)		.33
**Intercept^e^**	1.32 (0.01)	NA		2.1 (0.03)		<.001
***R* ^2 f^**	0.04	NA		0.15		NA

Abbreviations: SE, standard error; NA, not applicable.

^a^ Estimates are weighted to account for the sampling design.

^b^ Comparisons were made by *t* test to the reference group in each category.

^c^ Other categories of general health status were excellent, very good, and good.

^d^ Reference category is participants who live in a nonmetropolitan area.

^e^ Refers to the intercept in the ordinary least squares (OLS) model.

^f^ The amount of variability in psychological distress symptoms that is explained by the OLS model.

The results of the Wald test showed that the overall effect of the interaction between discrimination and race/ethnicity on distress was significant (*F* = 4.49, *P* = .003). Likewise, test of the individual interaction coefficients showed that blacks (β = −.06, *t* = −2.48, *P* = .02) and people of other races/ethnicities (β = −0.07, *t* = −2.99; *P* = .004) were significantly different from whites in the association of racial discrimination with psychological distress. Holding constant all other variables, for a 1-unit increase in discrimination, the K6 score of whites, Hispanics, blacks, Asians, and people of other races/ethnicities increased on average by 0.15, 0.13, 0.09, 0.13, and 0.08, respectively (Table 3). The effect of discrimination on distress depended on race/ethnicity; this effect was stronger for whites than for blacks and people of other races/ethnicities (Figure).

**Table 3:** Interaction Among Discrimination, Distress, and Race/Ethnicity in California Adults, California Health Interview Survey, 2005 (N = 26,291,280)^a^

Interaction Term	Unstandardized Coefficient (95% CI)	*z* Score	*P* Value^b^
Non-Hispanic white × discrimination	0.15 (0.14–0.17)	18.58	<.001
Hispanic × discrimination	0.13 (0.11–0.16)	10.41	<.001
Non-Hispanic black × discrimination	0.09 (0.04–0.14)	3.50	<.001
Non-Hispanic Asian × discrimination	0.13 (0.10–0.16)	8.04	<.001
Other race/ethnicity × discrimination	0.08 (0.03–0.13)	3.30	.001

Abbreviation: CI, confidence interval.

^a^ Estimates are weighted to account for the sampling design.

^b^ Calculated by using a *z* test.

**Figure:**
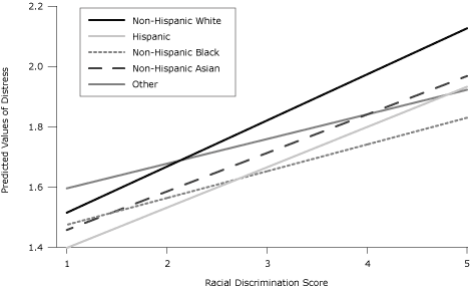
Predicted psychological distress by race/ethnicity and discrimination among California adults, California Health Interview Survey, 2005 (N = 26,291,280)), based on a multiple regression model with controls for age, sex, education level, employment status, self-reported general health status, nativity and citizenship status, English use and proficiency, ability to understand doctor at last visit, and geographic location.

**Table:** 

Levels of Discrimination					
**Racial/Ethnic Group**	**1 (Never)**	**2 (Rarely)**	**3 (Sometimes)**	**4 (Often)**	**5 (All the time)**
**Non-Hispanic White**	1.51529	1.668071	1.820853	1.973634	2.126415
**Hispanic**	1.398301	1.531878	1.665455	1.799032	1.93261
**Non-Hispanic Black**	1.475078	1.5638	1.652522	1.741244	1.829966
**Non-Hispanic Asian**	1.457925	1.585503	1.713081	1.840659	1.968237
**Other**	1.595937	1.677618	1.759299	1.84098	1.922661

## Discussion

My analysis of whites, Hispanics, blacks, Asians, and people of other races/ethnicities in California yielded 3 major findings: 1) reports of encountering racial discrimination differed significantly among racial/ethnic groups, and racial/ethnic minorities reported higher levels of discrimination than whites; 2) self-reported discrimination was significantly related to psychological distress symptoms, after controlling for race/ethnicity, age, sex, education level, employment status, general health status, nativity and citizenship status, English use and proficiency, ability to understand doctor at last visit, and geographic location; and 3) the effect of self-reported discrimination on psychological distress symptoms was modified by race/ethnicity and was weaker for blacks and people of other races/ethnicities than for whites.

The finding that all 5 racial/ethnic minority groups reported higher levels of discrimination than whites is consistent with studies that have conceptualized the experience of discrimination as a stressor in the lives of stigmatized people (22,23). Stress theory postulates that discrimination places racial/ethnic minority groups (ie, socially stratified groups rather than the proportion of the population) at the lower end of the racial hierarchy, and as a result of this social placement, minorities may encounter additional stressors through the interaction between race and class (24). The second finding of a linear relationship between discrimination and distress has been reported in other studies, which found that experiences of discrimination are related to poorer mental health status and quality of life (25–28).

The finding that the effect of discrimination on symptoms of psychological distress was stronger for whites than for blacks and persons of other races is contrary to our original hypothesis but is consistent with the notion that experiences of discrimination may affect the health of whites more adversely than that of blacks. Kessler (20) documented a similar pattern for the relationship between stressful life events and psychological distress for nonwhites (mainly blacks) and people of low socioeconomic status. Both groups were more exposed to stress, but similar stressful encounters had more of a negative effect on whites and people with high socioeconomic status than on nonwhites and people with low socioeconomic status. Williams and colleagues (21) adjusted for race-related stress (major discrimination and everyday experiences of unfairness) and found that blacks reported significantly lower levels of psychological distress when compared with whites. Likewise, my results indicate that blacks and people of other races/ethnicities reported greater levels of racial discrimination than whites; although whites experienced discrimination less often, it had more of an adverse effect on their distress levels than it had on blacks.

Kessler (20) suggested some possible reasons why experiences of discrimination affected whites more adversely. First, blacks may be more accustomed to dealing with stressful experiences because they are more frequently exposed to adversity. Hence, they seldom have an extreme emotional response to a new stressor. In essence, objective stressors such as discrimination may have a different subjective meaning for blacks than for whites. Second, compared with whites, blacks may respond with greater emotional flexibility (ie, one’s emotional response to stress), which may facilitate recovery. In addition, blacks may also use different coping mechanisms, such as religious involvement, that some have argued may further reduce the negative effects of stress (29).

My results also indicate that blacks reported, on average, significantly more discrimination than did all other racial groups, whereas Hispanics reported significantly less than all other racial groups except whites; the difference between members of other races and Asians was not significant. This difference in level of perceived discrimination may give blacks resiliency, which may explain blacks’ mental health advantage relative to whites in my study.

My study has several limitations. First, the study used cross-sectional data from a single year, which does not allow evaluation of causal associations. Several prospective studies have found that reports of discrimination predict subsequent illness and changes in mental health status (30,31). For instance, Schulz and colleagues (30) examined the longitudinal relationship between self-reported everyday discrimination and health among 343 African American women in Detroit, Michigan. They found that everyday encounters with discrimination (eg, being treated with less courtesy or respect) were causally associated with poorer mental and physical health outcomes, independent of the effects of education and income. Thus, longitudinal data would be more appropriate for determining causality and the temporal changes in psychological distress symptoms.

Another limitation is that my study used a self-reported, single-item discrimination measure, which may introduce response and measurement biases. Dohrenwend (32) noted that recall bias, unreliability of recall, and criterion and construct validity are all potential problems of self-reported measures. The discrimination measure used for this study did not focus on a long recall period or retrospective reports; rather, it assessed the frequency of perceived unfair or bad treatment based on one’s race or ethnicity. The use of a single-item measure may not completely capture the full range of discrimination experiences (26,33). Research has shown that severe events are recalled better than less severe ones, so a single-item measure may not accurately capture the effect of severe discriminatory incidents (34). Using more comprehensive scales that prompt respondents to recall specific instances, such as those that occur at school or in the work place, may better capture experiences of discrimination that may otherwise be missed by using only a single global question. Hence, the effect of discrimination on distress may have been stronger among racial/ethnic minorities if I had used full questionnaires (21,35,36). Future research using more accurate measures of discrimination will likely produce better response and recall, minimizing reporting and measurement biases.

Finally, the generalizability of my study findings is limited by the low response rate of CHIS and our analyses of only California residents. The CHIS low response rate introduces potential sampling bias whereby nonresponders may differ in key ways from responders. CHIS respondents, however, match the demographic profile of California residents based on the 2008 US Census. Additionally, the CHIS rate is comparable to the response rates of other telephone surveys in California, such as the 2005 BRFSS. Although these external data temper concerns over response bias, some skepticism should be maintained about the results, given the poor response rate. Furthermore, it is unclear how generalizable my results are outside of California. Future research using a national sample could test whether these findings hold across populations and geography.

My study had several strengths. First, I controlled for many sociodemographic and immigration-related factors, including the use of a standard Centers for Disease Control and Prevention measure of self-rated health, which allowed for comparison with other studies (37). Second, my study was not restricted to specific populations such as adolescents or college students; instead, it was based on a probability sample of all California adults. Finally, I used a large sample, which allowed me to disaggregate my analyses and examine the effect of self-reported discrimination on psychological distress among 5 racial/ethnic groups.

I found that reports of discrimination varied significantly by each racial/ethnic group. My analyses further suggested that the relationship between discrimination and psychological distress was conditional on race/ethnicity. Future studies should elucidate the causal mechanism of discrimination on psychological distress and investigate this relationship in other racial/ethnic populations, including immigrants.
